# Detection and Genetic Characterization of Seoul Virus in Liver Tissue Samples From *Rattus norvegicus* and *Rattus tanezumi* in Urban Areas of Southern China

**DOI:** 10.3389/fvets.2021.748232

**Published:** 2021-12-13

**Authors:** Wenqiao He, Jiaqi Fu, Yuqi Wen, Mingji Cheng, Yun Mo, Qing Chen

**Affiliations:** Department of Epidemiology, School of Public Health, Guangdong Provincial Key Laboratory of Tropical Disease Research, Southern Medical University, Guangzhou, China

**Keywords:** liver, rats, Seoul virus, prevalence, genetic diversity

## Abstract

Rodents are important hosts of hantaviruses, and lungs and kidneys are known to be the preferred organs of these viruses. Recently, hantaviruses were detected in liver samples from wild rodents in Hungary and the United States, and feeder rats in the Netherlands. However, few studies have detected hantaviruses in the liver of rats from China. In this study, hantaviruses were investigated in liver samples from *R. norvegicus* and *R. tanezumi* trapped in urban areas of southern China. A total of 461 *R. norvegicus* and 64 *R. tanezumi* were trapped. Using a pan-hantavirus PCR method, hantaviruses were detected in liver, lung, and serum samples from these animals. About 7.43% of liver samples were positive for Seoul virus (SEOV). The detection rate of SEOV in liver samples from *R. norvegicus* (8.24%) was higher than that from *R. tanezumi* (1.56%), suggesting the predominant role of *R. norvegicus* in the transmission of SEOV in urban areas of China. Three *R. norvegicus* had SEOV RNA in their liver samples but not in their lung samples, suggesting that the liver might be one of the targeted organs of SEOV. The first full SEOV protein-coding sequences (CDS) of the S and M segments, and partial CDS of the L segment from *R. tanezumi* were amplified. Several full and partial CDS of the S, M, and L segments from *R. norvegicus* were also obtained. The SEOV sequences obtained from different animals were highly similar, suggesting the cross-species transmission potential of SEOV between *R. norvegicus* and *R. tanezumi*.

## Introduction

Hantaviruses belong to the subfamily *Mammantavirinae* within the family *hantaviridae* (1). They are enveloped, single-stranded, negative-sense RNA viruses, with genomes composed of small (S), medium (M), and large (L) segments (2). The L segment encodes the viral RNA-dependent RNA polymerase (RdRp), while the M and S segments encode the two envelope proteins (Gn and Gc) and the nucleocapsid protein (N), respectively (2). To date, more than 50 hantavirus species have been recognized (3). Some of these viruses are not pathogenic, such as the Prospect Hill virus (4), but at least 24 species of hantaviruses are able to cause human diseases (5). Hantaviruses mainly cause two diseases in humans: hemorrhagic fever with renal syndrome (HFRS), and hantavirus cardiopulmonary syndrome (HCPS) (6).

Literature describing hantavirus infections in China dates back to the 12th century (7). Even today, hantavirus infections remain a serious health concern in China, accounting for 90% of HFRS cases globally (8). The most common hantavirus species responsible for causing HFRS are the Dobrava–Belgrade, Hantaan, Seoul, and Puumala viruses (9).

Rodents are reservoirs of HFRS-associated hantaviruses, and each hantavirus associated with a distinct rodent host species (10). *Myodes* rodent species usually host Puumala viruses, *Apodemus* rodent species commonly host Dobrava–Belgrade viruses, and *Rattus* and *Apodemus* rodent species usually host Seoul virus (SEOV) and Hantaan virus (HTNV) (11). The population densities, community composition and species distributions of rodents have important influences on HFRS transmission (12, 13). In China, SEOV and HTNV are the two dominant hantavirus species that cause HFRS in urban and agricultural areas, respectively (14). *Rattus norvegicus* (Norway rat) is the predominant reservoir of SEOV in urban residential areas in China (8). Other *Rattus* species rodents, such as *Rattus tanezumi* (Asian house rat), are also prevalent in urban areas of southern China, and are also hosts of SEOV (15).

Understanding the natural infection of hantaviruses to reservoir hosts is important for clarifying the pathogenesis and transmission routes of the viruses. The main transmission routes of hantaviruses are usually direct contact with rodents or inhalation of contaminated aerosols from excretions or secretions of infected animals (16). Most of the studies to date have reported hantaviruses in lung and kidney samples from rodents (17, 18), with only a few studies focusing on hantaviruses in other organs, such as the liver (1, 19). A recent study reported the detection of Dobrava–Belgrade virus, Puumala virus, and Tula virus in liver tissue samples from *Apodemus, Myodes*, and *Microtus* rodent species in Hungary (19). SEOV has been detected in liver tissue samples from feeder rats in the Netherlands and from wild *R. norvegicus* in New York (1, 20). Furthermore, histopathologic changes associated with SEOV infection have primarily been observed in liver samples from feeder rats (1). In addition to HFRS, SEOV can also cause other diseases in humans, such as mild hepatitis (21). Therefore, it is important to investigate SEOV in the liver of naturally infected wild reservoir hosts to understand its tropism and pathogenesis. In this study, SEOV in liver tissue samples from urban *Rattus* species rodents in southern China were investigated.

## Materials and Methods

### Sample Collection

**Figure 1:**
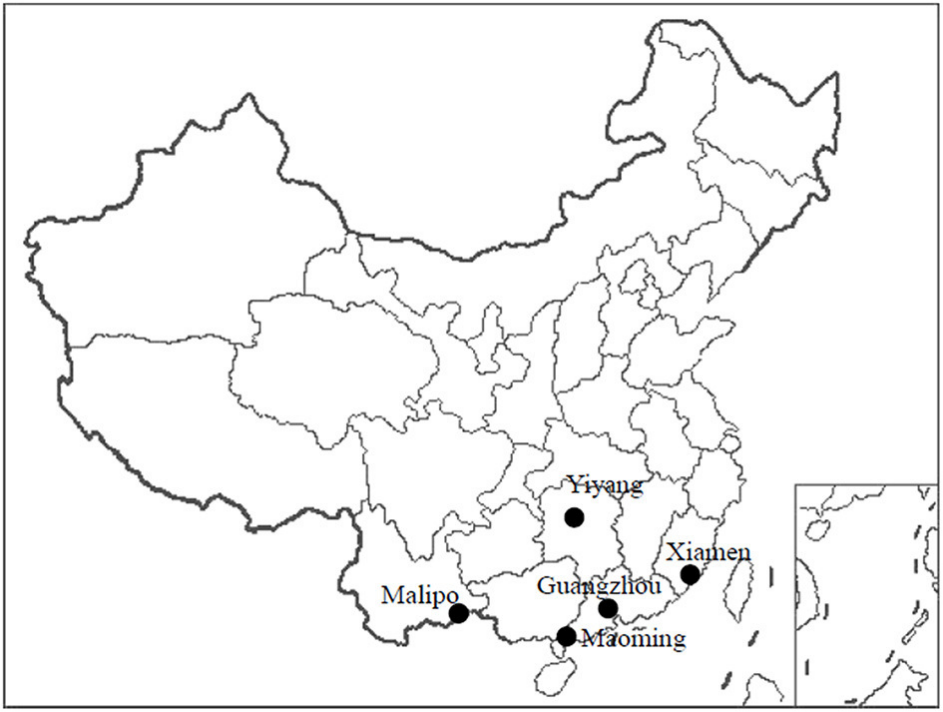
Locations of the animal trapping sites in China. Map source: https://image.so.com/view?q=%E4%B8%AD%E5%9B%BD%E5%9C%B0%E5%9B%BE&src=srp&correct=%E4%B8%AD%E5%9B%BD%E5%9C%B0%E5%9B%BE&ancestor=list&cmsid=50712c46325496ee4c7fbfbded18f06e&cmras=0&cn=0&gn=0&kn=50&crn=0&bxn=20&fsn=130&cuben=0&pornn=0&manun=50&adstar=0&clw=233#id=812433445ba11b77cba553308beab1d9&currsn=0&ps=136&pc=136.

Animals were trapped in five regions in southern China (Guangzhou City and Maoming City in Guangdong Province, Yiyang City in Hunan Province, Xiamen City in Fujian Province, and Malipo County in Yunan Province) between 2014 and 2018 (Figure 1 and Table 1). All animals were captured near human residences using cage traps. The trapped animals were anesthetized by 3% diethyl ether inhalation, with the dosage adjusted according to their heart rate, respiratory frequency, corneal reflection, and the extremity muscle tension. Trained personnel wore filtering facepiece respirators, chemical safety goggles, anti-static uniforms, and chemical protective gloves to protect them from diethyl ether. Blood was drawn by cardiac puncture and centrifuged to obtain serum samples. Animals were then executed by cervical dislocation. Liver tissue and lung tissue samples were obtained and soaked in RNAlater (Invitrogen, Carlsbad, CA, USA). All of the samples were stored at −80°C prior to processing. The species of each animal was identified by sequencing cytochrome B (*cytB*) gene (22).

**Table 1:** Detection of Seoul virus in liver tissue samples from the trapped animals.

**Species**	**Hunan Province**	**Fujian Province**	**Yunnan Province**	**Guangdong** **Province**		**Total**
	**Yiyang City**	**Xiamen City**	**Malipo County**	**Guangzhou City**	**Maoming City**	
*Rattus norvegicus*	2.27 (2/88)	8.57 (3/35)	3.92 (2/51)	13.43 (27/201)	4.65 (4/86)	8.24 (38/461)
*Rattus tanezumi*	5.26 (1/19)	0 (0/24)	0 (0/1)	0 (0/13)	0 (0/7)	1.56 (1/64)
Total	2.80 (3/107)	5.08 (3/59)	3.85 (2/52)	12.62 (27/214)	4.30 (4/93)	7.43 (39/525)

### Extraction of Nucleic Acid and Hantavirus Detection

Total RNA and DNA were extracted from ~20 mg of liver tissue samples or 200 μL aliquot of serum samples using the MiniBEST Viral RNA/DNA Extraction Kit (TaKaRa, Kusatsu, Japan). Given the previous report that lung was the most preferred organ for the detection of SEOV (1), total RNA and DNA were also extracted from ~20 mg of lung tissue samples from animals with SEOV-positive liver tissue samples using the MiniBEST Viral RNA/DNA Extraction Kit (TaKaRa).

A nested PCR assay designed based on a conserved region within the L segment of hantaviruses was used to detect currently known and possible novel members within the genus *Orthohantavirus* (23). The amplified products were separated on a 1.5% agarose gel, and positive samples were sent to the Beijing Genomics Institute (Shenzhen, China) for sequencing. All the PCR screening sequences were aligned with the sequences in the NCBI nucleotide sequence (NCBI NT) database (https://www.ncbi.nlm.nih.gov/nucleotide) by using BLASTN.

### Genomic Sequencing

Based on the reference sequences (L segment: KX079474.1, KM948596.1; M segment: KM948597.1, M34882.1; S segment: NC_005236.1, EF192308.1), primers were designed to amplify the near full-length genome of SEOV (Additional File 1). After sequencing all of the fragments, the amplified sequences were aligned with the sequences in the NCBI nucleotide sequence (NCBI NT) database (https://www.ncbi.nlm.nih.gov/nucleotide) by using BLASTN. Fragments that did not represent SEOV were excluded. Then the Lasergene SeqMan software (DNASTAR, Madison, WI, USA) was used to assemble the sequences with reference-based approach (assemble method: pro assemble). Unassembled singletons were verified by amplification using different primers that designed based on the SEOV sequences to ensure they represent SEOV.

### Phylogenetic Analysis

All of the screening sequences, and the full and partial protein-coding sequences (CDS) obtained in this study were aligned with hantavirus genomic sequences from GenBank (http://www.ncbi.nlm.nih.gov/genbank/) using the ClustalW multiple sequence alignment program in MEGA (version 7.0; Oxford Molecular, Cambridge, UK). Phylogenetic trees were constructed based on the PCR screening sequences, and full and partial CDS of the S, M, and L fragments via MrBayes (version 3.2; https://www.nbisweden.github.io/MrBayes/). Using the GTR + G + I nucleotide substitution matrix, two million Markov chain Monte Carlo (MCMC) iterations were sampled every 100 steps to obtain 20,000 trees, with the burn-in generally being 25% of the tree replicates (24). The identities of the amino acid and nucleotide sequences were estimated using the Sequence Identity Matrix program in BioEdit (version 7.2.5; https://file.org/free-download/bioedit). Recombination events were investigated using SimPlot (version 3.5.1; https://sray.med.som.jhmi.edu/SCRoftware/SimPlot/).

### Selective Pressure Analysis

Selective pressure analyses were performed based on the full and partial CDS of the S, M, and L segments. The neighbor-joining method (Kimura 2-parameter model) in MEGA (version 7.0; Oxford Molecular, Cambridge, UK) was used to construct phylogenetic trees. The free-ratio model in CODEML in EasyCodeML (version 1.21; https://github.com/BioEasy/EasyCodeML) was used to calculate the *dN* (nonsynonymous substitution), *dS* (synonymous substitution), and ω*-*values (*dN/dS*) for each branch (25). The ω-values of >1, 1, or <1 indicating positive selection, neutral evolution, or negative selection, respectively. Site models (M0, M1a, M2a, M3, M7, M8, and M8a) were applied to detect potential selection among sites. We also used likelihood-ratio tests (LRTs) to assess these models and the Bayes Empirical Bayes (BEB) to evaluate the posterior probability of positive selection sites (26). Positive selection sites were identified as those with a BEB score larger than 0.95.

### Statistical Analysis

Statistical analysis was performed using the Statistical Product and Service Solutions software (SPSS, version 13.0; IBM, Armonk, NY, USA). Descriptive statistics (Crosstabs) were used to assess detection rates. Chi-square tests were performed to test differences in detection rates across different animals and different sampling locations. *P* < 0.05 was considered to be statistically significant.

### Ethical Guidelines

The study protocol was approved by the Animal Ethics and Welfare Committee of the School of Public Health, Southern Medical University and adhered to the guidelines for the Rules for the Implementation of Laboratory Animal Medicine (1998) from the Ministry of Health, China. All surgical procedures were performed under anesthesia in efforts to minimize the suffering of the animals. Endangered or protected animal species were not included in this study.

## Results

### Detection of SEOV in Liver, Serum, and Lung Samples From Urban *Rattus* Species Rodents

From 2014 to 2018, a total of 525 *Rattus* species rodents were trapped, including 461 *Rattus norvegicus* and 64 *Rattus tanezumi* (Figure 1 and Table 1). Viral RNA and DNA were extracted from 525 liver tissue samples, while only 190 serum samples (more than 200 μL) were sufficient for nucleic acid extraction.

All of the PCR screening sequences obtained in our study belonged to SEOV (accession numbers: OL364860–OL364936). A total of 7.43% (39/525) of the liver tissue samples were positive for SEOV, with the detection rates of 8.24% (38/461) and 1.56% (1/64) in *R. norvegicus* and *R. tanezumi*, respectively (χ^2^ = 3.647, *P* > 0.05; Table 1). *R. norvegicus* trapped in Guangzhou City had the highest detection rate of SEOV (13.43%), followed by *R. norvegicus* trapped in Xiamen City (8.57%) and Maoming City (4.65%). All of the SEOV-positive serum samples were from *R. norvegicus* (1.76%, 3/170; Table 2), and the liver tissue samples from these three animals were also positive for SEOV.

**Table 2:** Detection of Seoul virus in serum samples from the trapped animals.

**Species**	**Fujian Province**	**Yunnan Province**	**Guangdong** **Province**		**Total**
	**Xiamen City**	**Malipo County**	**Guangzhou City**	**Maoming City**	
*Rattus norvegicus*	5.88 (1/17)	0 (0/24)	2.56 (2/78)	0 (0/51)	1.76 (3/170)
*Rattus tanezumi*	0 (0/10)	–	0 (0/5)	0 (0/5)	0 (0/20)
Total	3.70 (1/27)	0 (0/24)	2.41 (2/83)	0 (0/56)	1.58 (3/190)

Lung tissue samples from rats with SEOV-positive liver samples were used to detect SEOV RNA. Aside from the missing lung tissue sample from animal MM86, a total of 38 lung tissue samples were obtained. Among these samples, three lung tissue samples tested negative for SEOV (Table 3).

**Table 3:** Seoul virus in the lung tissue and serum samples from animals with Seoul virus-positive liver tissue samples.

	**XM4**	**XM47**	**XM49**	**MM17**	**MM23**	**MM32**	**MM86**	**YN11**	**YN45**	**YY27**	**YY40**	**YY55**	**GZ15**
Serum samples	N	P	N	–	N	N	–	–	–	–	–	–	–
Lung tissue samples	P	P	P	N	P	P	–	P	P	P	P	P	P
	**GZ45**	**GZ47**	**GZ48**	**GZ50**	**GZ52**	**GZ53**	**GZ59**	**GZ213**	**GZ219**	**GZ223**	**GZ224**	**GZ263**	**GZ264**
Serum samples	–	–	–	–	–	–	–	–	–	–	–	–	–
Lung tissue samples	P	P	P	P	P	N	P	P	P	P	P	N	P
	**GZ325**	**GZ326**	**GZ329**	**GZ330**	**GZ333**	**GZ431**	**GZ437**	**GZ462**	**GZ464**	**GZ466**	**GZ471**	**GZ473**	**GZ488**
Serum samples	P	N	N	N	N	–	N	N	N	–	N	P	N
Lung tissue samples	P	P	P	P	P	P	P	P	P	P	P	P	P

*P, Positive in the detection of Seoul virus*.

*N, Negative in the detection of Seoul virus*.

*-, Serum samples that are not sufficient for nucleic acid extraction*.

### Genomic Amplification and Phylogenetic Analysis

**Figure 2:**
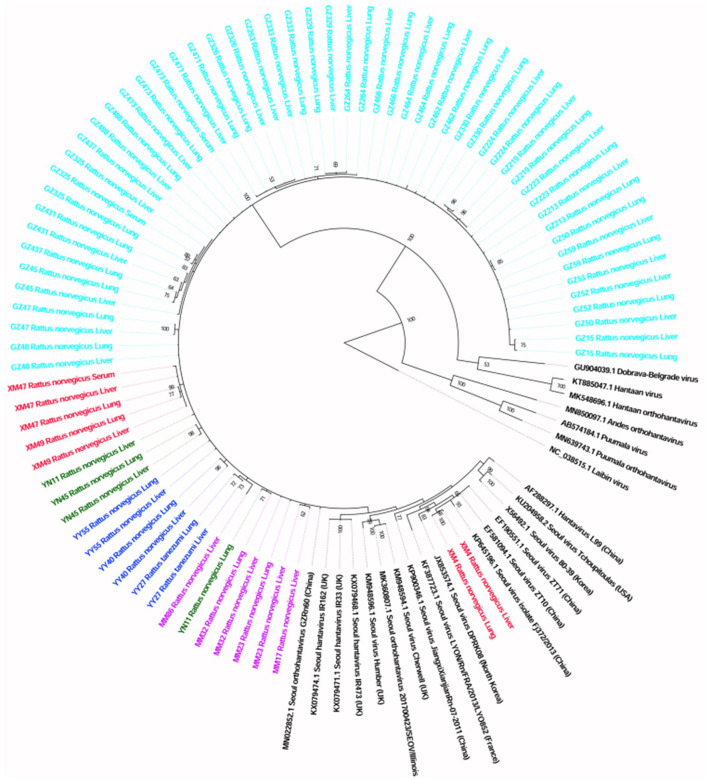
Phylogenetic tree constructed using PCR screening nucleotide sequences of Seoul virus obtained from rats. Twenty-two representative orthohantavirus sequences were included for comparison, and one Laibin virus sequence was set as the outgroup. The percentages of the posterior probability (PP) values are indicated.

The sequences detected in different samples from the same animal were highly similar (>99% at the nucleotide level; Additional File 2), and the sequences from animals trapped in the same sampling location usually clustered together (Figure 2).

**Figure 3:**
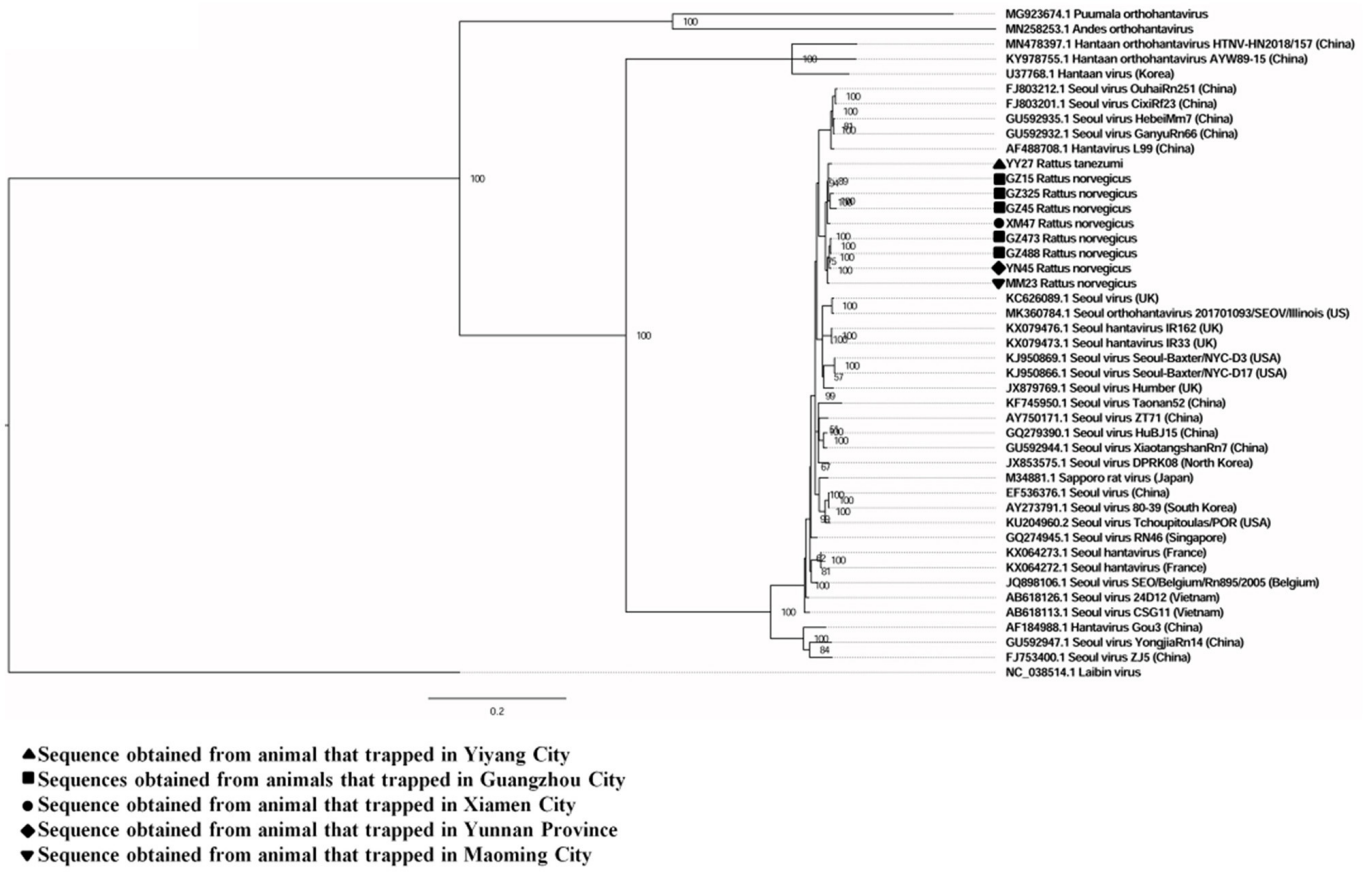
Phylogenetic tree constructed using nucleotide sequences of the Seoul virus S gene obtained from rats. Thirty-five representative orthohantavirus sequences were included for comparison, and one Laibin virus sequence was set as the outgroup. The percentages of the posterior probability (PP) values are indicated.

**Figure 4:**
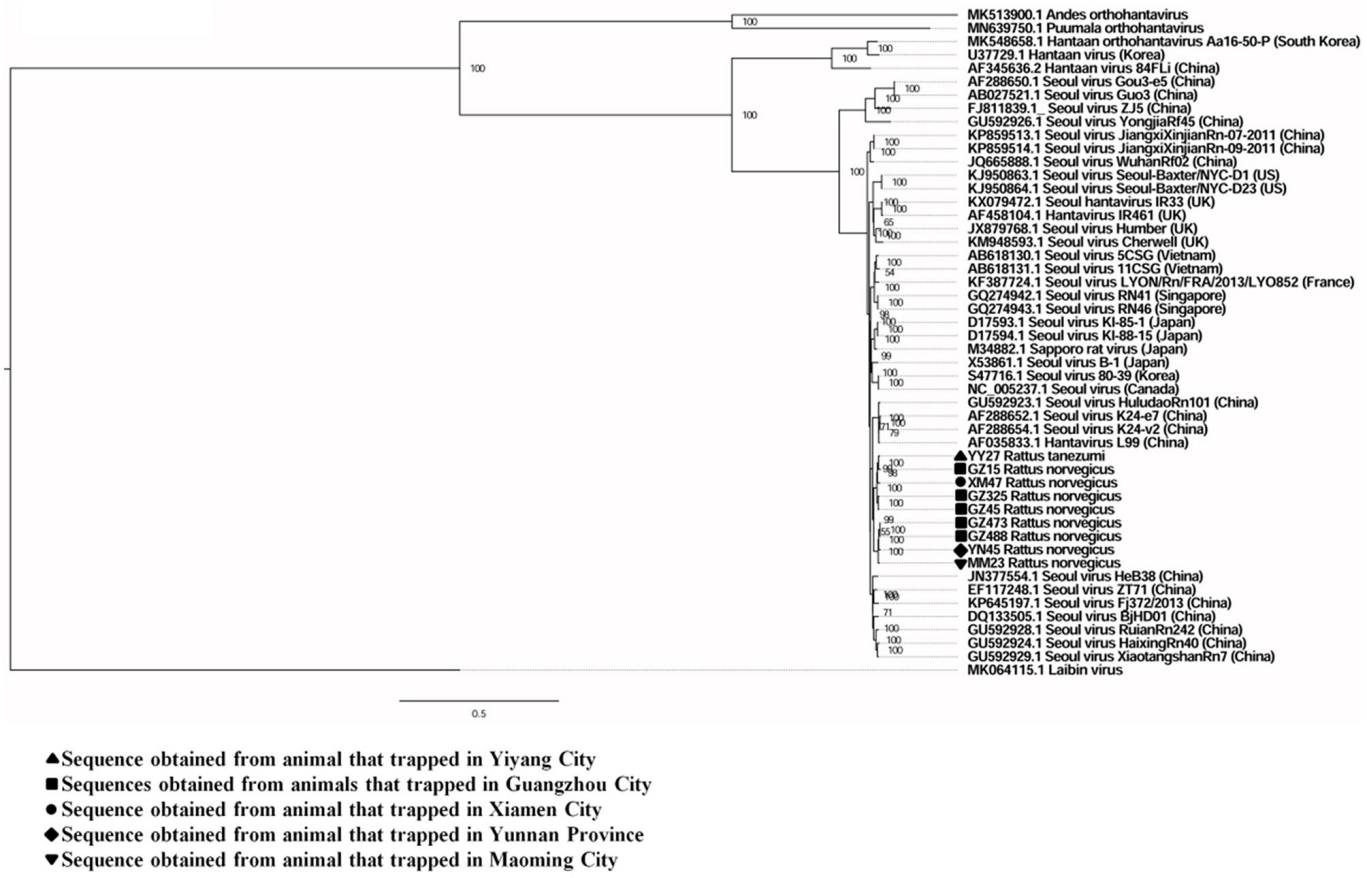
Phylogenetic tree constructed using nucleotide sequences of the Seoul virus M gene obtained from rats. Forty representative orthohantavirus sequences were included for comparison, and one Laibin virus sequence was set as the outgroup. The percentages of the posterior probability (PP) values are indicated.

**Figure 5:**
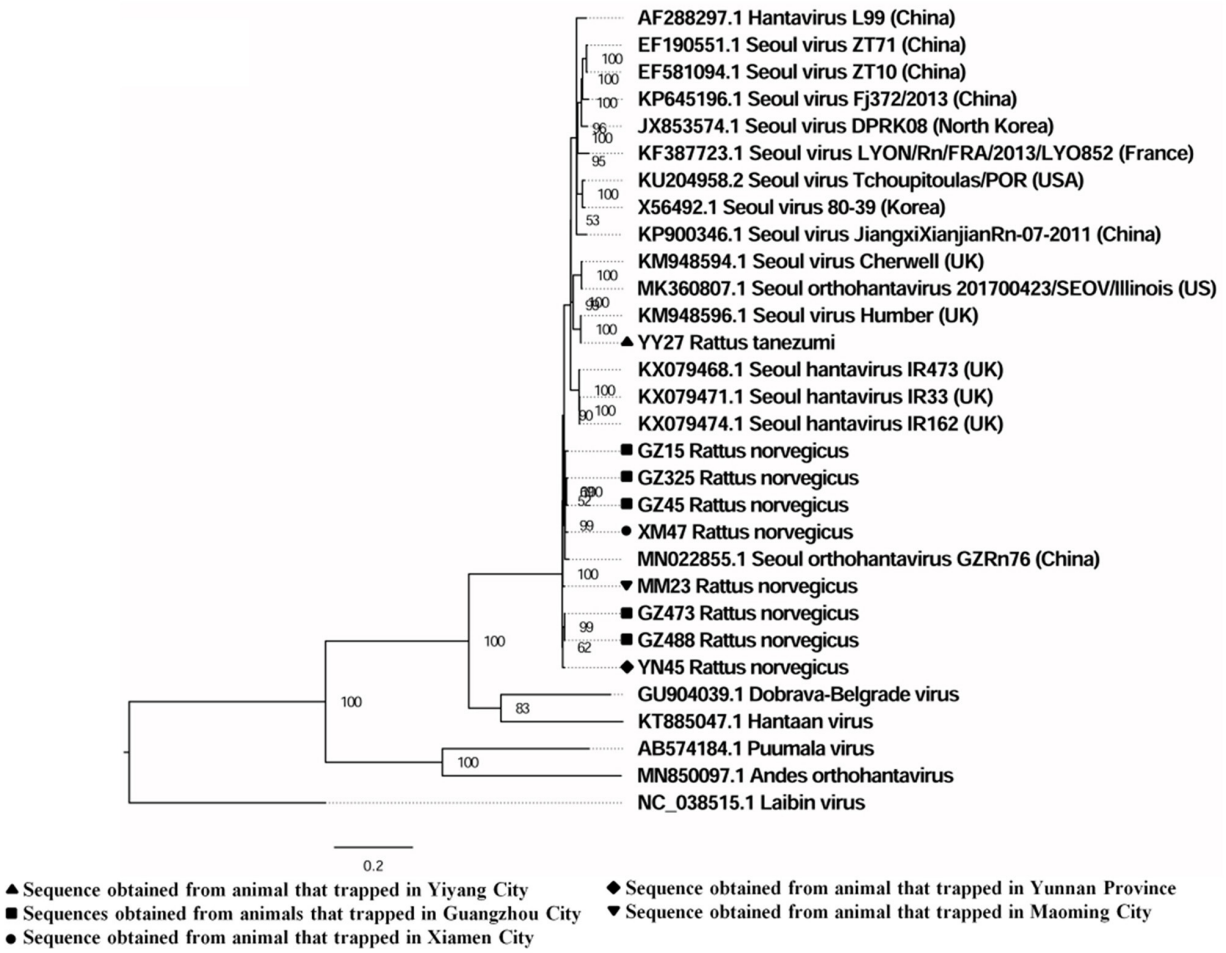
Phylogenetic tree constructed using nucleotide sequences of the Seoul virus L gene obtained from rats. Twenty representative orthohantavirus sequences were included for comparison, and one Laibin virus sequence was set as the outgroup. The percentages of the posterior probability (PP) values are indicated.

A total of six full and two partial CDS of the S segment (GenBank accession numbers: MZ031957-MZ031964), eight partial CDS of the M segment (GenBank accession numbers: MZ031948-MZ031955), and eight partial CDS of the L segment were obtained from *R. norvegicus* (GenBank accession numbers: MZ031938- MZ031944, MZ031946). In addition, the first full CDS of the S (GenBank accession number: MZ031956) and M (GenBank accession number: MZ031947) segments, and partial CDS of the L (GenBank accession number: MZ031945) segment were also amplified from *R. tanezumi*. High level of similarity was found between the full and partial CDS obtained from *R. norvegicus* and *R. tanezumi*, indicating the cross-species transmission potential of SEOV between these species (Figures 3–5 and Additional Files 3–5). Most of the CDS clustered with SEOV sequences obtained from rats in China, while the partial CDS of the L segment from *R. tanezumi* was most similar to one SEOV sequence detected in *R. norvegicus* from the United Kingdom. Further analyses revealed no recombination events in the sequences obtained in our study.

### Selective Constraints During the Evolution of SEOV

Selective pressure analysis was performed based on the full and partial CDS of the S, M, and L segments. The ω-values of three segments (S, M, and L) varied and all of them were <1 (ω = 0.00997, 0.01846, and 0.01302, respectively), suggesting the negative selective pressure on all of the SEOV genes. The M gene had the largest ω-value (0.01846), indicating a greater selective pressure on this gene. Site model analysis found no amino acid sites with signals of positive selection.

## Discussion

Only a few studies have investigated SEOV in liver tissue samples from urban *Rattus* species rodents in China (27, 28). In this study, SEOV was detected in liver tissue samples from urban *R. norvegicus* and *R. tanezumi* in southern China.

A nested-PCR assay that can detect currently known and possible novel members within the genus *Orthohantavirus* was used for hantavirus screening, and all of the PCR screening sequences detected in our study belonged to SEOV. This is consistent with the results of previous studies, suggesting that SEOV is one of the prevalent hantavirus species in residential habitats in China (29, 30). However, different methods are still needed to confirm the presence of other hantavirus species within the samples.

In this study, SEOV RNA was detected in liver tissue samples from *R. norvegicus* and *R. tanezumi*. The presence of SEOV RNA in lung tissue samples from *R. norvegicus* and *R. tanezumi* was also reported (15). The above suggests that these animals are natural hosts of SEOV in southern China. The highest detection rate was found in *R. norvegicus*, which is consistent with the results of previous studies and indicates the predominant role of *R. norvegicus* in the transmission of SEOV (29, 31).

Detection of SEOV RNA in liver tissue, lung tissue, and serum samples from *Rattus* species rodents in our study confirms that SEOV has multiple targeted organs in naturally infected reservoir hosts. Previous studies have shown that the lung is the most preferred organ for detecting SEOV in feeder rats and wild *R. norvegicus* (1, 20). Interestingly, we found that three *R. norvegicus* with SEOV-positive liver tissue samples had SEOV-negative lung tissue samples, which is inconsistent with the results of previous studies (1, 20). In addition, histopathologic changes associated with SEOV infection have been primarily found in the liver of rats (1). It seems that the liver is one of the targeted organs of SEOV in naturally infected wild *Rattus* species rodents. Our research group is also investigating SEOV in the lung tissue samples from rats. About 200 lung tissue samples from rats have been investigated (detailed results will be reported in the future). Among these animals, three *R. norvegicus* with SEOV-positive lung tissue samples had SEOV-negative liver tissue samples, suggesting that the positivity within one tissue sample doesn't always correspond to the positivity in other tissue samples. When investigating SEOV in animals, different organs should be tested to fully understand the prevalence of viral infection and the risk of disease transmission. More studies are needed to investigate the tropism and pathogenesis of SEOV in its naturally infected reservoir hosts.

In this study, sequences from animals trapped in same sampling location usually clustered together, which might be explained by the limited movement of the reservoir hosts (32). Geographic clustering of SEOV has also been found in other studies (33). The full and partial CDS from *R. norvegicus* and *R. tanezumi* were highly similar, suggesting the cross-species transmission potential of SEOV between *R. norvegicus* and *R. tanezumi*. The partial CDS of the L segment from *R. tanezumi* obtained in this study was most similar to an L segment detected in *R. norvegicus* from the United Kingdom (34). Previous studies have reported that the species *R. norvegicus* probably originated from northern China and migrated to other countries along with human activities (29, 35). These findings suggest that human activities and cross-species transmission potential of SEOV might responsible for the worldwide distribution of SEOV and the similarity between SEOV in different species of reservoir hosts from different countries. However, further study is needed to confirm the cross-species transmission potential of SEOV among rats.

Despite previous studies have showed that the recombination events appear to be common in hantaviruses (15, 36), no recombination event was found in our study. The recombination of SEOV remains to be studied in more large-scale studies. In this study, negative selection is the principal evolutionary force on SEOV, which is consistent with the results of previous studies (33).

There were several limitations in this study. Firstly, we did not identify the age and sex of the animals, which might affect the prevalence of SEOV. Secondly, due a lack of pathological descriptions, we could not confirm the pathogenicity of SEOV in animals. Thirdly, only one *R. tanezumi* was infected with SEOV in this study, large scale study is needed to investigate SEOV in *R. tanezumi* and confirm the cross-species transmission potential of SEOV among rats. Fourthly, since we did not collect kidney samples, we were unable to include kidney analyses in this study. In addition, SEOV was only investigated in partial serum samples and the lung tissue samples of individuals with SEOV detection in the liver tissue samples. Further study is needed to investigate the tissue tropism of SEOV in rats.

## Conclusions

In this study, SEOV in liver tissue samples from urban *Rattus* species rodents in China was investigated. In addition, liver was found to be one of the targeted organs of SEOV in naturally infected wild *Rattus* species rodents, which expands our knowledge of natural SEOV infection in its reservoir hosts. In the future, more rigorous studies will be needed to investigate the tissue tropism of SEOV in its naturally infected reservoir hosts. When monitoring SEOV infection in animals, it is important to include liver tissue samples to provide comprehensive information about the viral prevalence.

## Data Availability Statement

The datasets supporting the conclusions of this article are included within the article and its additional files. All PCR screening sequences and full and partial CDS of SEOV are available in the NCBI database (Accession Nos: OL364860-OL364936, and MZ031938-MZ031964).

## Ethics Statement

The study protocol was approved by the Animal Ethics and Welfare Committee of the School of Public Health, Southern Medical University and adhered to the guidelines for the Rules for the Implementation of Laboratory Animal Medicine (1998) from the Ministry of Health, China. All surgical procedures were performed under anesthesia in efforts to minimize the suffering of the animals. Endangered or protected species were not included in this study.

## Author Contributions

WH and QC conceived of the project and QC obtained the funding. WH and QC contributed to the writing of the paper. WH and JF performed the experiment. WH, YW, and MC analyzed the data. WH, JF, YW, MC, and YM collected the samples. All of the authors have read and approved the manuscript for publication.

## Funding

This work was supported by the Key-Area Research and Development Program of Guangdong Province (no. 2018B020241002) and the National Natural Science Foundation of China (no. 81373051). The funders had no role in the study design, data collection and analysis, decision to publish, or preparation of the manuscript.

## Conflict of Interest

The authors declare that the research was conducted in the absence of any commercial or financial relationships that could be construed as a potential conflict of interest.

## Publisher's Note

All claims expressed in this article are solely those of the authors and do not necessarily represent those of their affiliated organizations, or those of the publisher, the editors and the reviewers. Any product that may be evaluated in this article, or claim that may be made by its manufacturer, is not guaranteed or endorsed by the publisher.

## Abbreviations

BEB: Bayes Empirical Bayes
CDS: protein-coding sequences
*cytB*: cytochrome B
HCPS: hantavirus cardiopulmonary syndrome
HFRS: hemorrhagic fever with renal syndrome
HTNV: Hantaan virus
LRTs: likelihood-ratio tests
MCMC: Markov chain Monte Carlo
RdRp: RNA-dependent RNA polymerase
*R. norvegicus*: *Rattus norvegicus*
*R. tanezumi*: *Rattus tanezumi*
SEOV: Seoul virus
SPSS: Statistical Product and Service Solutions software.
