# Supplementary material for: Detection and Genetic Characterization of Seoul Virus in Liver Tissue Samples From Rattus norvegicus and Rattus tanezumi in Urban Areas of Southern China
Source: Front Vet Sci. 2021 Dec 13;8:748232. doi: 10.3389/fvets.2021.748232 (PMC8710597; doi:10.3389/fvets.2021.748232)
Supplement: Supplementary file 1 [file Table_1.pdf]

Additional file 1. Primers for viral amplification

Primer	Orientation	Sequence (5'-3')	Target fragment (base pairs)
S-F1	Sense (first/second round)	TAGTAGTAGACTCCCTAAAGAG	928
S-R928	Antisense (second round)	GGTGACTCAATATGTTCAAC	
S-R979	Antisense (first round)	CATGTTGGTGGACACCTATC	
S-F894	Sense (first/second round)	CAGGCAACATTCAAAGGATGC	824
S-R1717	Antisense (second round)	GAAACAACTTAATGAGGTAGTATA	
S-R1746	Antisense (first round)	AGTAGTAKRCTCCCTAAAAAGAC	
M-F1	Sense (first/second round)	TAGTAGTAGACTCCGCAAG	1034
M-R1034	Antisense (second round)	CTAAATATGAAACGACCATCTTC	
M-R1143	Antisense (first round)	CTGGACTGCCTCATAGTATC	
M-F681	Sense (first/second round)	CATCAGAAAGGGAATACATATAAG	724
MR1404	Antisense (second round)	CAGTGAAAAGAGACTTGTAATAG	
MR1452	Antisense (first round)	CTGGAACACACARCTCAATAG	
M-F1245	Sense (first/second round)	CTCCAATGTGTCTGGTGTC	1251
M-R2495	Antisense (second round)	CACAAAGCAATCATTCATATC	
M-R2545	Antisense (first round)	GAGAGAACTTAGATACAGTC	
M-F2364	Sense (first/second round)	CAGGTTGTACTGCTTGTGGATT	961
M-R3324	Antisense (second round)	GATTATACCCATAACCCATTCAC	
M-R3587	Antisense (first round)	CAATAAAAGATTGGGAGATAG	
L- F1	Sense (first/second round)	TAGTAGTAGACTCCGGAAGAG	1028
L-R1028	Antisense (second round)	CCATATCATGTGCAAGAATAG	
L-R1099	Antisense (first round)	GGGCTAAATTGGATTCTAGG	
L-F935	Sense (first/second round)	CAGCATACAAACCTGCAACTATAC	1331
L-R2266	Antisense (second round)	CTTTGTTGCCCATTCTACAGTTTC	
L-R2305	Antisense (first round)	CAACTAACATCTCACCATACTTATC	
L-F2227	Sense (first/second round)	TTGCATGGGAATGTGAATGAAG	992
L-R3219	Antisense (second round)	CAAATAATGATGAGCACTTGTTC	
L-R3282	Antisense (first round)	GCAGTCYAATTCTGGAAATAAC	
L-F3136	Sense (first/second round)	GAACCTCATGTCAGAGACTTC	984
L-R4119	Antisense (second round)	CATATGTAGGATGCTGTTCACTC	
L-R4209	Antisense (first round)	GCATAGTCATAGCTGTTATAATG	
L-F3989	Sense (first/second round)	CTGGTGAGACATTTCAGCATG	1128
L-R5117	Antisense (second round)	ATTGGCTTGGAGTTGTAACACTG	
L-R5121	Antisense (first round)	CTATTGGCTTGGAGTTGTAAC	
L-F5076	Sense (first/second round)	GTGTGCAGCATTGATGATTGC	1316
L-R6392	Antisense (second round)	CTGGAATCACATCCTCAGGTA	
L-R6457	Antisense (first round)	GAAACTGAATCCTGTGGAAATAG	

Additional file 2. Nucleotide and amino acid identities (%) of PCR screening sequences in different samples from the same animal.

	XM4[b]	XM47[a]	XM49[b]	MM23[b]	MM32[b]	YN11[b]	YN45[b]	YY27[b]	YY40[b]	YY55[b]	GZ15[b]	GZ4[b]
Nucleotide identity	99.6	99.0-99.6	99.3	99.3	99.6	99.3	99.3	99.6	99.6	99.6	99.3	99.3
Amino acid identity	100	100	100	100	100	100	100	100	100	100	100	100
	GZ47[b]	GZ48[b]	GZ50[b]	GZ52[b]	GZ59[b]	GZ213[b]	GZ219[b]	GZ223[b]	GZ224[b]	GZ263	GZ264[b]	GZ325[a]
Nucleotide identity	100	99.6	99.3	99.6	99.6	99.6	99.6	99.3	99.6	-	99.3	99.0-99.6
Amino acid identity	100	100	100	100	100	100	100	99.0	100	-	100	100
	GZ326[b]	GZ329[b]	GZ330[b]	GZ333[b]	GZ431[b]	GZ437[b]	GZ462[b]	GZ464[b]	GZ466[b]	GZ471[b]	GZ473[a]	GZ488[b]
Nucleotide identity	99.3	99.3	99.3	99.6	99.0	99.3	99.0	99.0	99.0	99.3	99.6-100	99.0
Amino acid identity	100	100	100	100	100	100	100	100	100	100	100	100

a: Animals that have Seoul virus-positive liver tissue, lung tissue and serum samples.

b: Animals that have Seoul virus-positive liver tissue and lung tissue samples.

Additional file 3. Nucleotide and amino acid identities of the near full-length S segments

	YY27	GZ15	GZ325	GZ473	MM23	GZ45	XM47	YN45	GZ488	NC_038514.1	MG923674.1	MN258253.1	MN478397.1	MK360784.1	KY978755.1	U37768.1	JX853575.1	JX879769.1
YY27	ID	0.991 (1.000)	0.982 (0.990)	0.979 (0.993)	0.979 (0.993)	0.986 (0.995)	0.990 (0.997)	0.986 (1.000)	0.986 (1.000)	0.560 (0.500)	0.620 (0.628)	0.617 (0.644)	0.726 (0.827)	0.965 (0.995)	0.740 (0.822)	0.743 (0.832)	0.968 (0.997)	0.967 (1.000)
GZ15	0.991 (1.000)	ID	0.982 (0.990)	0.978 (0.993)	0.980 (0.993)	0.986 (0.995)	0.991 (0.997)	0.985 (1.000)	0.986 (1.000)	0.563 (0.500)	0.621 (0.628)	0.617 (0.644)	0.727 (0.827)	0.964 (0.995)	0.742 (0.822)	0.744 (0.832)	0.967 (0.997)	0.968 (1.000)
GZ325	0.982 (0.990)	0.982 (0.990)	ID	0.982 (0.997)	0.985 (0.997)	0.981 (0.990)	0.982 (0.988)	0.975 (0.990)	0.976 (0.990)	0.554 (0.495)	0.616 (0.623)	0.613 (0.637)	0.721 (0.820)	0.956 (0.986)	0.736 (0.815)	0.735 (0.825)	0.959 (0.988)	0.958 (0.990)
GZ473	0.979 (0.993)	0.978 (0.993)	0.982 (0.997)	ID	0.992 (1.000)	0.972 (0.988)	0.979 (0.990)	0.988 (0.993)	0.992 (0.993)	0.556 (0.495)	0.615 (0.625)	0.611 (0.639)	0.721 (0.820)	0.957 (0.988)	0.731 (0.815)	0.737 (0.825)	0.958 (0.990)	0.956 (0.993)
MM23	0.979 (0.993)	0.980 (0.993)	0.985 (0.997)	0.992 (1.000)	ID	0.976 (0.988)	0.981 (0.990)	0.986 (0.993)	0.986 (0.993)	0.558 (0.495)	0.616 (0.625)	0.610 (0.639)	0.721 (0.820)	0.958 (0.988)	0.734 (0.815)	0.736 (0.825)	0.963 (0.990)	0.959 (0.993)
GZ45	0.986 (0.995)	0.986 (0.995)	0.981 (0.990)	0.972 (0.988)	0.976 (0.988)	ID	0.986 (0.993)	0.979 (0.995)	0.980 (0.995)	0.558 (0.500)	0.618 (0.623)	0.612 (0.639)	0.730 (0.825)	0.961 (0.990)	0.740 (0.820)	0.742 (0.829)	0.963 (0.993)	0.964 (0.995)
XM47	0.990 (0.997)	0.991 (0.997)	0.982 (0.988)	0.979 (0.990)	0.981 (0.990)	0.986 (0.993)	ID	0.987 (0.997)	0.986 (0.997)	0.563 (0.500)	0.619 (0.628)	0.614 (0.644)	0.727 (0.829)	0.964 (0.993)	0.743 (0.825)	0.742 (0.834)	0.968 (0.995)	0.968 (0.997)
YN45	0.986 (1.000)	0.985 (1.000)	0.975 (0.990)	0.988 (0.993)	0.986 (0.993)	0.979 (0.995)	0.987 (0.997)	ID	0.996 (1.000)	0.564 (0.500)	0.617 (0.628)	0.616 (0.644)	0.724 (0.827)	0.964 (0.995)	0.742 (0.822)	0.745 (0.832)	0.966 (0.997)	0.963 (1.000)
GZ488	0.986 (1.000)	0.986 (1.000)	0.976 (0.990)	0.992 (0.993)	0.986 (0.993)	0.980 (0.995)	0.986 (0.997)	0.996 (1.000)	ID	0.562 (0.500)	0.616 (0.628)	0.614 (0.644)	0.728 (0.827)	0.965 (0.995)	0.739 (0.822)	0.745 (0.832)	0.965 (0.997)	0.964 (1.000)
NC_038514.1	0.560 (0.500)	0.563 (0.500)	0.554 (0.495)	0.556 (0.495)	0.558 (0.495)	0.558 (0.500)	0.563 (0.500)	0.564 (0.500)	0.562 (0.500)	ID	0.576 (0.519)	0.561 (0.524)	0.569 (0.516)	0.559 (0.500)	0.569 (0.511)	0.571 (0.513)	0.558 (0.502)	0.557 (0.500)

MG923674.1	0.620 (0.628)	0.621 (0.628)	0.616 (0.623)	0.615 (0.625)	0.616 (0.625)	0.618 (0.623)	0.619 (0.628)	0.617 (0.628)	0.616 (0.628)	0.576 (0.519)	ID	0.681 (0.736)	0.619 (0.609)	0.611 (0.632)	0.622 (0.602)	0.608 (0.609)	0.611 (0.630)	0.618 (0.628)
MN258253.1	0.617 (0.644)	0.617 (0.644)	0.613 (0.637)	0.611 (0.639)	0.610 (0.639)	0.612 (0.639)	0.614 (0.644)	0.616 (0.644)	0.614 (0.644)	0.561 (0.524)	0.681 (0.736)	ID	0.636 (0.648)	0.617 (0.644)	0.618 (0.644)	0.631 (0.646)	0.618 (0.646)	0.617 (0.644)
MN478397.1	0.726 (0.827)	0.727 (0.827)	0.721 (0.820)	0.721 (0.820)	0.721 (0.820)	0.730 (0.825)	0.727 (0.829)	0.724 (0.827)	0.728 (0.827)	0.569 (0.516)	0.619 (0.609)	0.636 (0.648)	ID	0.731 (0.827)	0.842 (0.983)	0.860 (0.976)	0.724 (0.829)	0.732 (0.827)
MK360784.1	0.965 (0.995)	0.964 (0.995)	0.956 (0.986)	0.957 (0.988)	0.958 (0.988)	0.961 (0.990)	0.964 (0.993)	0.964 (0.995)	0.965 (0.995)	0.559 (0.500)	0.611 (0.632)	0.617 (0.644)	0.731 (0.827)	ID	0.742 (0.822)	0.742 (0.832)	0.961 (0.993)	0.972 (0.995)
KY978755.1	0.740 (0.822)	0.742 (0.822)	0.736 (0.815)	0.731 (0.815)	0.734 (0.815)	0.740 (0.820)	0.743 (0.825)	0.742 (0.822)	0.739 (0.822)	0.569 (0.511)	0.622 (0.602)	0.618 (0.644)	0.842 (0.983)	0.742 (0.822)	ID	0.853 (0.967)	0.743 (0.825)	0.742 (0.822)
U37768.1	0.743 (0.832)	0.744 (0.832)	0.735 (0.825)	0.737 (0.825)	0.736 (0.825)	0.742 (0.829)	0.742 (0.834)	0.745 (0.832)	0.745 (0.832)	0.571 (0.513)	0.608 (0.609)	0.631 (0.646)	0.860 (0.976)	0.742 (0.832)	0.853 (0.967)	ID	0.740 (0.834)	0.742 (0.832)
JX853575.1	0.968 (0.997)	0.967 (0.997)	0.959 (0.988)	0.958 (0.990)	0.963 (0.990)	0.963 (0.993)	0.968 (0.995)	0.966 (0.997)	0.965 (0.997)	0.558 (0.502)	0.611 (0.630)	0.618 (0.646)	0.724 (0.829)	0.961 (0.993)	0.743 (0.825)	0.740 (0.834)	ID	0.963 (0.997)
JX879769.1	0.967 (1.000)	0.968 (1.000)	0.958 (0.990)	0.956 (0.993)	0.959 (0.993)	0.964 (0.995)	0.968 (0.997)	0.963 (1.000)	0.964 (1.000)	0.557 (0.500)	0.618 (0.628)	0.617 (0.644)	0.732 (0.827)	0.972 (0.995)	0.742 (0.822)	0.742 (0.832)	0.963 (0.997)	ID

Amino acid identity is showed in bracket.
NC_038514.1: Laibin virus sequence.
MG923674.1: Puumala orthohantavirus sequence.
MN258253.1: Andes orthohantavirus sequence
MN478397.1, KY978755.1, U37768.1: Hantaan virus sequences.
MK360784.1, JX853575.1, JX879769.1: Seoul virus sequences.

Additional file 4. Nucleotide and amino acid identities of the near full-length M segments

	YY27	GZ325	GZ473	GZ488	GZ15	MM23	GZ45	XM47	YN45	KP645197.1	JX879768.1	KM948593.1	NC_005237.1	MK513900.1	MN639750.1	MK548658.1	AF345636.2	U37729.1	MK064115.1
YY27	ID	0.985 (0.995)	0.968 (0.994)	0.968 (0.994)	0.990 (0.995)	0.969 (0.993)	0.986 (0.995)	0.985 (0.990)	0.968 (0.995)	0.957 (0.993)	0.950 (0.989)	0.947 (0.992)	0.956 (0.988)	0.573 (0.537)	0.588 (0.536)	0.718 (0.767)	0.716 (0.762)	0.720 (0.770)	0.541 (0.450)
GZ325	0.985 (0.995)	ID	0.968 (0.999)	0.968 (0.999)	0.984 (0.998)	0.969 (0.998)	0.995 (0.998)	0.985 (0.993)	0.969 (0.998)	0.959 (0.998)	0.952 (0.991)	0.947 (0.995)	0.958 (0.992)	0.574 (0.536)	0.586 (0.537)	0.720 (0.767)	0.717 (0.762)	0.721 (0.770)	0.538 (0.451)
GZ473	0.968 (0.994)	0.968 (0.999)	ID	0.999 (1.000)	0.969 (0.997)	0.991 (0.997)	0.968 (0.997)	0.968 (0.992)	0.991 (0.997)	0.954 (0.999)	0.947 (0.990)	0.941 (0.994)	0.952 (0.991)	0.577 (0.535)	0.588 (0.537)	0.720 (0.766)	0.718 (0.761)	0.723 (0.769)	0.541 (0.452)
GZ488	0.968 (0.994)	0.968 (0.999)	0.999 (1.000)	ID	0.969 (0.997)	0.991 (0.997)	0.968 (0.997)	0.968 (0.992)	0.990 (0.997)	0.954 (0.999)	0.947 (0.990)	0.941 (0.994)	0.952 (0.991)	0.577 (0.535)	0.588 (0.537)	0.721 (0.766)	0.719 (0.761)	0.724 (0.769)	0.541 (0.452)
GZ15	0.990 (0.995)	0.984 (0.998)	0.969 (0.997)	0.969 (0.997)	ID	0.969 (0.996)	0.984 (0.998)	0.984 (0.993)	0.967 (0.996)	0.955 (0.996)	0.949 (0.991)	0.945 (0.995)	0.955 (0.990)	0.575 (0.537)	0.588 (0.536)	0.720 (0.767)	0.717 (0.762)	0.722 (0.770)	0.541 (0.451)
MM23	0.969 (0.993)	0.969 (0.998)	0.991 (0.997)	0.991 (0.997)	0.969 (0.996)	ID	0.969 (0.996)	0.969 (0.991)	0.990 (0.996)	0.957 (0.996)	0.950 (0.991)	0.944 (0.993)	0.953 (0.990)	0.577 (0.537)	0.587 (0.539)	0.720 (0.767)	0.718 (0.762)	0.723 (0.770)	0.540 (0.450)

GZ45	0.986 (0.995)	0.995 (0.998)	0.968 (0.997)	0.968 (0.997)	0.984 (0.998)	0.969 (0.996)	ID	0.986 (0.993)	0.968 (0.996)	0.959 (0.996)	0.952 (0.991)	0.947 (0.995)	0.957 (0.991)	0.573 (0.535)	0.585 (0.535)	0.720 (0.766)	0.717 (0.761)	0.721 (0.769)	0.538 (0.450)
XM47	0.985 (0.990)	0.985 (0.993)	0.968 (0.992)	0.968 (0.992)	0.984 (0.993)	0.969 (0.991)	0.986 (0.993)	ID	0.967 (0.991)	0.955 (0.991)	0.947 (0.987)	0.945 (0.990)	0.954 (0.986)	0.571 (0.533)	0.586 (0.536)	0.718 (0.767)	0.716 (0.760)	0.720 (0.768)	0.540 (0.450)
YN45	0.968 (0.995)	0.969 (0.998)	0.991 (0.997)	0.990 (0.997)	0.967 (0.996)	0.990 (0.996)	0.968 (0.996)	0.967 (0.991)	ID	0.954 (0.996)	0.947 (0.989)	0.941 (0.993)	0.953 (0.990)	0.578 (0.535)	0.587 (0.536)	0.719 (0.767)	0.718 (0.762)	0.723 (0.770)	0.541 (0.450)
KP645197.1	0.957 (0.993)	0.959 (0.998)	0.954 (0.999)	0.954 (0.999)	0.955 (0.996)	0.957 (0.996)	0.959 (0.996)	0.955 (0.991)	0.954 (0.996)	ID	0.951 (0.989)	0.945 (0.993)	0.957 (0.990)	0.576 (0.535)	0.586 (0.537)	0.713 (0.767)	0.717 (0.762)	0.719 (0.770)	0.541 (0.452)
JX879768.1	0.950 (0.989)	0.952 (0.991)	0.947 (0.990)	0.947 (0.990)	0.949 (0.991)	0.950 (0.991)	0.952 (0.991)	0.947 (0.987)	0.947 (0.989)	0.951 (0.989)	ID	0.965 (0.990)	0.949 (0.984)	0.574 (0.537)	0.589 (0.536)	0.715 (0.764)	0.718 (0.758)	0.718 (0.767)	0.539 (0.450)
KM948593.1	0.947 (0.992)	0.947 (0.995)	0.941 (0.994)	0.941 (0.994)	0.945 (0.995)	0.944 (0.993)	0.947 (0.995)	0.945 (0.990)	0.941 (0.993)	0.945 (0.993)	0.965 (0.990)	ID	0.948 (0.988)	0.574 (0.537)	0.586 (0.538)	0.712 (0.763)	0.715 (0.758)	0.713 (0.767)	0.541 (0.452)
NC_005237.1	0.956 (0.988)	0.958 (0.992)	0.952 (0.991)	0.952 (0.991)	0.955 (0.990)	0.953 (0.990)	0.957 (0.991)	0.954 (0.986)	0.953 (0.990)	0.957 (0.990)	0.949 (0.984)	0.948 (0.988)	ID	0.575 (0.533)	0.587 (0.535)	0.718 (0.766)	0.718 (0.761)	0.718 (0.769)	0.535 (0.450)
MK513900.1	0.573 (0.537)	0.574 (0.536)	0.577 (0.535)	0.577 (0.535)	0.575 (0.537)	0.577 (0.537)	0.573 (0.535)	0.571 (0.533)	0.578 (0.535)	0.576 (0.535)	0.574 (0.537)	0.574 (0.537)	0.575 (0.533)	ID	0.668 (0.667)	0.580 (0.540)	0.581 (0.540)	0.578 (0.541)	0.542 (0.466)
MN639750.1	0.588 (0.536)	0.586 (0.537)	0.588 (0.537)	0.588 (0.537)	0.588 (0.536)	0.587 (0.539)	0.585 (0.535)	0.586 (0.536)	0.587 (0.536)	0.586 (0.537)	0.589 (0.536)	0.586 (0.538)	0.587 (0.535)	0.668 (0.667)	ID	0.576 (0.544)	0.583 (0.546)	0.575 (0.545)	0.542 (0.461)
MK548658.1	0.718 (0.767)	0.720 (0.767)	0.720 (0.766)	0.721 (0.766)	0.720 (0.767)	0.720 (0.767)	0.720 (0.766)	0.718 (0.767)	0.719 (0.767)	0.713 (0.767)	0.715 (0.764)	0.712 (0.763)	0.718 (0.766)	0.580 (0.540)	0.576 (0.544)	ID	0.839 (0.962)	0.942 (0.984)	0.548 (0.455)
AF345636.2	0.716 (0.762)	0.717 (0.762)	0.718 (0.761)	0.719 (0.761)	0.717 (0.762)	0.718 (0.762)	0.717 (0.761)	0.716 (0.760)	0.718 (0.762)	0.717 (0.762)	0.718 (0.758)	0.715 (0.758)	0.718 (0.761)	0.581 (0.540)	0.583 (0.546)	0.839 (0.962)	ID	0.839 (0.960)	0.554 (0.452)
U37729.1	0.720 (0.770)	0.721 (0.770)	0.723 (0.769)	0.724 (0.769)	0.722 (0.770)	0.723 (0.770)	0.721 (0.769)	0.720 (0.768)	0.723 (0.770)	0.719 (0.770)	0.718 (0.767)	0.713 (0.767)	0.718 (0.769)	0.578 (0.541)	0.575 (0.545)	0.942 (0.984)	0.839 (0.960)	ID	0.542 (0.455)
MK064115.1	0.541 (0.450)	0.538 (0.451)	0.541 (0.452)	0.541 (0.452)	0.541 (0.451)	0.540 (0.450)	0.538 (0.450)	0.540 (0.450)	0.541 (0.450)	0.541 (0.452)	0.539 (0.450)	0.541 (0.452)	0.535 (0.450)	0.542 (0.466)	0.542 (0.461)	0.548 (0.455)	0.554 (0.452)	0.542 (0.455)	ID

Amino acid identity is showed in bracket.
KP645197.1, JX879768.1, KM948593.1, NC_005237.1: Seoul virus sequences.
MK513900.1: Andes orthohantavirus sequence.
MN639750.1: Puumala orthohantavirus sequence.
MK548658.1, AF345636.2, U37729.1: Hantaan virus sequences.
MK064115.1: Laibin virus sequence.

Additional file 5. Nucleotide and amino acid identities of the near full-length L segments

	KT885047.1	AB574184.1	GU904039.1	MN850097.1	NC_038515.1	KM948596.1	KM948594.1	KP645196.1	JX853574.1	EF190551.1	MK360807.1	GZ325	GZ473	GZ15	MM23	YN45	GZ45	XM47	YY27	GZ488
KT885047.1	ID	0.668 (0.690)	0.746 (0.852)	0.658 (0.684)	0.633 (0.641)	0.741 (0.851)	0.741 (0.852)	0.741 (0.852)	0.740 (0.853)	0.741 (0.850)	0.742 (0.852)	0.745 (0.853)	0.745 (0.855)	0.746 (0.854)	0.713 (0.831)	0.746 (0.854)	0.744 (0.852)	0.745 (0.854)	0.737 (0.847)	0.744 (0.854)
AB574184.1	0.668 (0.690)	ID	0.666 (0.698)	0.710 (0.769)	0.644 (0.648)	0.663 (0.679)	0.662 (0.681)	0.662 (0.682)	0.661 (0.681)	0.661 (0.680)	0.663 (0.681)	0.662 (0.682)	0.663 (0.682)	0.661 (0.683)	0.631 (0.647)	0.662 (0.683)	0.661 (0.680)	0.662 (0.683)	0.660 (0.678)	0.662 (0.682)

GU904039.1	0.746 (0.852)	0.666 (0.698)	ID	0.655 (0.683)	0.637 (0.646)	0.748 (0.855)	0.747 (0.856)	0.750 (0.856)	0.746 (0.857)	0.748 (0.853)	0.747 (0.856)	0.747 (0.857)	0.750 (0.858)	0.748 (0.857)	0.714 (0.861)	0.748 (0.857)	0.746 (0.855)	0.748 (0.857)	0.744 (0.851)	0.749 (0.857)
MN850097.1	0.658 (0.684)	0.710 (0.769)	0.655 (0.683)	ID	0.637 (0.638)	0.660 (0.674)	0.661 (0.676)	0.658 (0.675)	0.658 (0.676)	0.658 (0.675)	0.661 (0.675)	0.661 (0.676)	0.663 (0.676)	0.662 (0.677)	0.633 (0.642)	0.662 (0.675)	0.660 (0.675)	0.663 (0.676)	0.656 (0.672)	0.661 (0.675)
NC_038515.1	0.633 (0.641)	0.644 (0.648)	0.637 (0.646)	0.637 (0.638)	ID	0.634 (0.637)	0.633 (0.638)	0.636 (0.638)	0.636 (0.639)	0.635 (0.636)	0.633 (0.638)	0.637 (0.638)	0.636 (0.638)	0.636 (0.638)	0.606 (0.606)	0.637 (0.638)	0.636 (0.638)	0.636 (0.639)	0.631 (0.637)	0.635 (0.637)
KM948596.1	0.741 (0.851)	0.663 (0.679)	0.748 (0.855)	0.660 (0.674)	0.634 (0.637)	ID	0.961 (0.990)	0.944 (0.989)	0.945 (0.989)	0.947 (0.986)	0.961 (0.989)	0.955 (0.989)	0.955 (0.991)	0.956 (0.990)	0.909 (0.940)	0.954 (0.990)	0.954 (0.988)	0.955 (0.988)	0.992 (0.992)	0.952 (0.988)
KM948594.1	0.741 (0.852)	0.662 (0.681)	0.747 (0.856)	0.661 (0.676)	0.633 (0.638)	0.961 (0.990)	ID	0.941 (0.989)	0.943 (0.989)	0.943 (0.986)	0.997 (0.999)	0.954 (0.990)	0.952 (0.991)	0.954 (0.990)	0.908 (0.941)	0.952 (0.990)	0.953 (0.989)	0.953 (0.988)	0.954 (0.982)	0.950 (0.988)
KP645196.1	0.741 (0.852)	0.662 (0.682)	0.750 (0.856)	0.658 (0.675)	0.636 (0.638)	0.944 (0.989)	0.941 (0.989)	ID	0.971 (0.994)	0.975 (0.991)	0.941 (0.989)	0.944 (0.992)	0.946 (0.994)	0.943 (0.993)	0.902 (0.943)	0.946 (0.992)	0.943 (0.991)	0.945 (0.991)	0.937 (0.981)	0.944 (0.991)
JX853574.1	0.740 (0.853)	0.661 (0.681)	0.746 (0.857)	0.658 (0.676)	0.636 (0.639)	0.945 (0.989)	0.943 (0.989)	0.971 (0.994)	ID	0.972 (0.991)	0.943 (0.988)	0.946 (0.992)	0.946 (0.994)	0.945 (0.993)	0.901 (0.943)	0.947 (0.993)	0.946 (0.991)	0.946 (0.991)	0.938 (0.981)	0.944 (0.991)
EF190551.1	0.741 (0.850)	0.661 (0.680)	0.748 (0.853)	0.658 (0.675)	0.635 (0.636)	0.947 (0.986)	0.943 (0.986)	0.975 (0.991)	0.972 (0.991)	ID	0.943 (0.985)	0.947 (0.989)	0.947 (0.991)	0.946 (0.990)	0.903 (0.940)	0.947 (0.990)	0.946 (0.988)	0.947 (0.988)	0.940 (0.978)	0.944 (0.988)
MK360807.1	0.742 (0.852)	0.663 (0.681)	0.747 (0.856)	0.661 (0.675)	0.633 (0.638)	0.961 (0.989)	0.997 (0.999)	0.941 (0.989)	0.943 (0.988)	0.943 (0.985)	ID	0.954 (0.989)	0.953 (0.990)	0.955 (0.989)	0.909 (0.940)	0.952 (0.989)	0.954 (0.988)	0.954 (0.987)	0.954 (0.981)	0.951 (0.987)
GZ325	0.745 (0.853)	0.662 (0.682)	0.747 (0.857)	0.661 (0.676)	0.637 (0.638)	0.955 (0.989)	0.954 (0.990)	0.944 (0.992)	0.946 (0.992)	0.947 (0.989)	0.954 (0.989)	ID	0.982 (0.996)	0.986 (0.996)	0.938 (0.947)	0.983 (0.996)	0.993 (0.996)	0.987 (0.996)	0.950 (0.983)	0.981 (0.996)
GZ473	0.745 (0.855)	0.663 (0.682)	0.750 (0.858)	0.663 (0.676)	0.636 (0.638)	0.955 (0.991)	0.952 (0.991)	0.946 (0.994)	0.946 (0.994)	0.947 (0.991)	0.953 (0.990)	0.982 (0.996)	ID	0.982 (0.997)	0.942 (0.948)	0.989 (0.998)	0.981 (0.995)	0.982 (0.995)	0.947 (0.984)	0.996 (0.996)
GZ15	0.746 (0.854)	0.661 (0.683)	0.748 (0.857)	0.662 (0.677)	0.636 (0.638)	0.956 (0.990)	0.954 (0.990)	0.943 (0.993)	0.945 (0.993)	0.946 (0.990)	0.955 (0.989)	0.986 (0.996)	0.982 (0.997)	ID	0.936 (0.946)	0.981 (0.995)	0.985 (0.995)	0.986 (0.995)	0.948 (0.982)	0.980 (0.995)
MM23	0.713 (0.831)	0.631 (0.647)	0.714 (0.861)	0.633 (0.642)	0.606 (0.606)	0.909 (0.940)	0.908 (0.941)	0.902 (0.943)	0.901 (0.943)	0.903 (0.940)	0.909 (0.940)	0.938 (0.947)	0.942 (0.948)	0.936 (0.946)	ID	0.943 (0.948)	0.937 (0.946)	0.938 (0.946)	0.904 (0.935)	0.944 (0.951)
YN45	0.746 (0.854)	0.662 (0.683)	0.748 (0.857)	0.662 (0.675)	0.637 (0.638)	0.954 (0.990)	0.952 (0.990)	0.946 (0.992)	0.947 (0.993)	0.947 (0.990)	0.952 (0.989)	0.983 (0.996)	0.989 (0.998)	0.981 (0.995)	0.943 (0.948)	ID	0.982 (0.995)	0.983 (0.995)	0.949 (0.984)	0.988 (0.996)
GZ45	0.744 (0.852)	0.661 (0.680)	0.746 (0.855)	0.660 (0.675)	0.636 (0.638)	0.954 (0.988)	0.953 (0.989)	0.943 (0.991)	0.946 (0.991)	0.946 (0.988)	0.954 (0.988)	0.993 (0.996)	0.981 (0.995)	0.985 (0.995)	0.937 (0.946)	0.982 (0.995)	ID	0.987 (0.995)	0.949 (0.982)	0.981 (0.995)
XM47	0.745 (0.854)	0.662 (0.683)	0.748 (0.857)	0.663 (0.676)	0.636 (0.639)	0.955 (0.988)	0.953 (0.988)	0.945 (0.991)	0.946 (0.991)	0.947 (0.988)	0.954 (0.987)	0.987 (0.996)	0.982 (0.995)	0.986 (0.995)	0.938 (0.946)	0.983 (0.995)	0.987 (0.995)	ID	0.949 (0.982)	0.982 (0.995)
YY27	0.737 (0.847)	0.660 (0.678)	0.744 (0.851)	0.656 (0.672)	0.631 (0.637)	0.992 (0.992)	0.954 (0.982)	0.937 (0.981)	0.938 (0.981)	0.940 (0.978)	0.954 (0.981)	0.950 (0.983)	0.947 (0.984)	0.948 (0.982)	0.904 (0.935)	0.949 (0.984)	0.949 (0.982)	0.949 (0.982)	ID	0.947 (0.983)
GZ488	0.744 (0.854)	0.662 (0.682)	0.749 (0.857)	0.661 (0.675)	0.635 (0.637)	0.952 (0.988)	0.950 (0.988)	0.944 (0.991)	0.944 (0.991)	0.944 (0.988)	0.951 (0.987)	0.981 (0.996)	0.996 (0.996)	0.980 (0.995)	0.944 (0.951)	0.988 (0.996)	0.981 (0.995)	0.982 (0.995)	0.947 (0.983)	ID

Amino acid identity is showed in bracket.
KT885047.1: Hantaan virus sequence.
AB574184.1: Puumala virus sequence.
GU904039.1: Dobrava-Belgrade virus sequence.

MN850097.1: Andes orthohantavirus virus sequence.
NC_038515.1: Laibin virus sequence.
KM948596.1, KM948594.1, KP645196.1, JX853574.1, EF190551.1, MK360807.1: Seoul virus sequences.
